# Exploring the Role of Coping Strategies on the Impact of Client Suicide: A Structural Equation Modeling Approach

**DOI:** 10.1177/00302228211073213

**Published:** 2022-01-31

**Authors:** Ruth Van der Hallen, Brian P. Godor

**Affiliations:** 1 Department of Psychology, Education & Child Studies, 6984 Erasmus University Rotterdam, Rotterdam, The Netherlands

**Keywords:** patient suicide, bereavement, clinicians, coping, therapy

## Abstract

Client suicide refers to cases where a mental health practitioner is exposed, affected, or bereaved by a client’s suicide and is known to have a profound impact on MHPs. The current study investigated the role of coping styles in understanding short- and long-term impact of client suicide. An international sample of 213 mental health practitioners who experienced a client suicide completed a survey on coping strategies (i.e., Brief-COPE) and the impact of traumatic events (i.e., impact of event scale-revised, long-term emotional impact scale and professional practice impact scale). Results indicate coping strategies explain 51% of the short-term, 64% of the long-term emotional and 55% of the long-term professional differences in impact of client suicide. Moreover, while an Avoidant coping style predicted more impact of client suicide, Positive coping and Humor predicted less impact of client suicide. Social Support coping did not predict impact of client suicide. Implications for both research and clinical practice are discussed.

Client suicide, used to refer to cases where a mental health practitioner (MHP) is exposed, affected, or bereaved by a client’s suicide, is not uncommon in clinical practice (Eagles et al., 2001). Research suggests about 20% of psychologists and 50% of psychiatrists experience client suicide (Chemtob et al., 1989), though incidence rates vary depending on the geographical area (e.g., Finlayson & Simmonds, 2018; Greenberg & Shefler, 2014; Rothes et al., 2013). Unsurprisingly, 97% of clinicians claim client suicide to be their greatest fear (Ellis & Patel, 2012).

In the immediate aftermath of client suicide, MHPs are reported to experience emotions of shock, disbelief, confusion, and denial (Kleespies et al., 1990) as well as feelings of distress, depression, anger at the client and/or agency/society, guilt, shame, failure, incompetence and a profound sense of responsibility (Ting et al., 2006). Research suggests approximately 50% of psychologists and social workers who have experienced client suicide go on to display post-traumatic stress symptoms (Dransart et al., 2014; Ting et al., 2006). Moreover, client suicide seems to elicit symptomatology comparable to what we see when one loses a parent (Chemtob et al., 1988; Greenberg & Shefler, 2014).

Looking at the long-term impact of client suicide, MHPs are likely to be subject to both far-reaching personal and professional consequences. Long-term *emotional* consequences may include depressive rumination, self-doubt, inadequacy, loneliness, isolation from their peers, sensitivity to signs of suicidal risk, and concern over one’s competence to treat (Alexander et al., 2000; Ellis & Patel, 2012; Hendin et al., 2000). Long-term *professional* consequences may include more attentiveness to legal matters of the profession, increased hours of supervision and intervision, vigilance and cautious when dealing with at-risk patients, or in general, for one to adopt a more conservative approach when treating suicidal clients (Alexander et al., 2000; Rothes et al., 2013; Ting et al., 2006). In addition, research suggests about 15% of MHPs considered early retirement following client loss (Alexander et al., 2000; Gulfi et al., 2010).

Individual differences regarding the impact of client suicide on MHPs have been the topic of investigation—in particular in relation to experience, age, and gender. Coping strategies, the cognitive and behavioral ways we try to manage the demands of stressful situations (Lazarus & Folkman, 1984), have not been explored in relation to how MHPs deal with client suicide. Interestingly, one’s coping style is known to impact the extent to which we are affected by trauma in general. Jeavons (2000) looked into coping strategies to predict psychological trauma following road accidents and found an emotion-focused coping style to be an important predictor of trauma at follow-up. Elmose and colleagues (2016) investigated the importance of coping following an explosion disaster and found self-blame, cognitive restructuring, and wishful thinking explain differences in symptomatology. Stratta and colleagues (2015) investigated to what extent coping and resilience would shape one’s psychological response following the 2009 earthquake in L’Aguila, Italy. Results indicated that resilience directly affected post-traumatic stress symptomatology (PTSS) —and that this relationship was mediated by differences in coping styles, explaining up to 30% of the variance in PTSS outcome.

Interestingly, the importance of coping in relation to losing a client to suicide has not been investigated previously. Although some research suggests MHPs may not feel as responsible and guilty when client suicide is dealt with in a suicide-accepting manner (Darden & Rutter, 2011; Sanders et al., 2008), which coping strategies could prove (mal)adaptive during traumatic bereavement, remains unclear. While coping with bereavement is different for each individual, there are likely common themes in dealing with the aftermath of client suicide that are worth unraveling (Gaffney & Hannigan, 2010). Yet, what general coping styles are adaptive versus maladaptive or beneficial versus detrimental in the case of MHPs who are dealing with the aftermath of client suicide, remains unclear. Say, however, one’s general coping style is predictive of the impact of client suicide, screening early career MHPs for at-risk coping styles and providing MHPs with psycho-education as to how coping and bereavement could be linked, may prove a valuable resource.

The current study aims to investigate to what extent one’s general coping style can help explain the short- and long-term impact of client suicide and, if so, which coping styles serve as protective versus risk factors to the bereavement process.

## Methods

### Participants

The study included 213 MHPs who experienced one or more client suicides. 53 identified as male (25%), 158 as female (72%) and 2 as non-binary (1%). Age ranged between 18 and 75, with 90% of participants aged between 18–55 years of age. Profession was indicated as psychologist (46%), psychiatric nurse (14%), psychiatrist (13%), counselor (10%), social worker (9%), or other (8%). The majority of the sample originated in Belgium (47%), Germany (18%), and the Netherlands (15%).

### Procedure

The present study is part of a larger research project looking into the impact of client suicide. Study protocols were in accordance with the ethical standards of the ethical committee of the Erasmus University of Rotterdam. Individual informed consent was obtained prior to participation. Participant recruitment was set up through the distribution of the survey via email, social media, or professional newsletters. Data was collected using a self-administered, online survey available in English, Dutch, and German. Note, the Brief-COPE and impact of event scale-revised (IES-R) are readily available in the abovementioned languages, the long-term emotional impact scale (LTEIS) and professional practice impact scale (PPIS) were back-translated by native speakers for the purpose of the current study (Tyupa, 2011). Participants were instructed that, in case that they had experienced more than one client suicide throughout their career, they should focus on the most distressing case and answer all questions with that client suicide in mind.

### Material

#### Brief-COPE

The Brief-COPE is an abbreviated version of the COPE (Carver et al., 1989; Carver, 1997) Inventory, a self-report questionnaire developed to assess a broad range of coping responses. The Brief-COPE includes 14 two-item subscales: (1) Use of emotional support, (2) Use of instrumental support, (3) Venting, (4) Active coping, (5) Planning, (6) Acceptance, (7) Positive Reframing, (8) Humor, (9) Religion, (10) Substance Use, (11) Behavioral disengagement, (12) Self-Distraction, (13) Self-Blame and (14) Denial. Each item is rated on a 4-point scale, ranging from “I haven’t been doing this at all” to “I’ve been doing this a lot.” Following EFA/CFA of the Brief-COPE, construct reliability was evaluated for our subscales; see Results, revealing acceptable Joreskog rho scores.

#### Impact of Event Scale-Revised

The IES-R is a revised version of the original IES (Creamer et al., 2003; Weiss & Marmar, 1997), a self-report questionnaire developed to assess the impact of a particular (traumatic) event in the 7 days following the event. The IES-R includes 22 items divided over 3 subscales: (1) Intrusion, (2) Hyperarousal, and (3) Avoidance. Each item is rated on a 5-point Likert scale, ranging from 0 “*Not at all*” to 4 “*Extremely*”. While the IES-R is not a diagnostic tool for PTSD in itself, the consensus is that sum scores between 24 and 32 suggest partial PTSD or at least some PTSD symptoms, sum scores between 33 and 38 suggest a PTSD diagnosis is probable, and sum scores of 39 or above suggest long-term impact (Creamer et al., 2003). Cronbach’s alpha as calculated for the current sample was α = .95, suggesting excellent scale reliability.

#### Long-Term Emotional Impact Scale

The Long-Term Emotional Impact Scale (LTEIS; Gulfi et al., 2010; Horn, 1994) is a self-report questionnaire developed to assess the long-term emotional impact of client suicide. The LTEIS includes 10 items, each referring to emotional consequences one may experience following client suicide. Items include experiencing increased concern, increased anxiety or increased helplessness when working with suicidal clients, experiencing guilt about client suicide, or experiencing increased sensitivity to signs of suicidal risk. Items are rated on a 5-point Likert scale, ranging from 1 “*Disagree*” to 5 “*Agree*”. Cronbach’s alpha as calculated for the current sample was α = .87, suggesting good scale reliability.

#### Professional Practice Impact Scale

The Professional Practice Impact Scale (PPIS; Gulfi et al., 2010, inspired by Henry et al., 2004) is a self-report questionnaire developed to assess the consequences of client suicide on one’s working practices. The PPIS includes nine items, each referring to professional consequences of client suicide commonly reported in the literature. Items include being more inclined to hospitalize suicidal clients, being more included to consult colleagues or supervisors, refusing to work with suicidal clients, or considering leaving the profession because of client suicide. Items are rated on a 5-point Likert scale, ranging from 1 “*Disagree*” to 5 “*Agree*”. Cronbach’s alpha as calculated for the current sample was α = 0.77, suggesting good scale reliability.

### Data Analysis

Statistical analyses were conducted using IBM SPSS Statistics 25.0 and AMOS 24.0 for Windows and include exploratory factor analysis (EFA), confirmatory factor analysis (CFA), and structural equation modeling (SEM). EFA analyses were performed in SPSS using principal axis factoring as an extraction method, with oblimin rotation and Kaiser normalization. To determine the best factor structure, the following aspects were taken into account: (i) minimum eigenvalues of 1, (ii) factor loadings 0.4 and above, (iii) inspection of the scree plot, and (iv) individual factors had to be conceptually coherent and reasonable (Ledesma et al., 2015).

Subsequently a CFA and SEM analyses were performed in AMOS using the maximum likelihood estimation method. Tests for multivariate normality revealed two variables with large kurtosis. Subsequently, a bootstrapping procedure was performed (*n* = 5000, CI = .95). All estimates were contained within the 95% confidence intervals. Global model fit was evaluated using the Comparative Fit Index (CFI; CFI ≥ .90), Root Mean Square Error of Approximation (RMSEA; 0.05 ≥ RMSEA ≤ 0.08) and Standardized Root Mean Square Residual (SRMS; ≥ .09) (Hu & Bentler, 1999; Savalei, 2018; 2021; Wang et al., 2018; Xia & Yang, 2019). Local fit was evaluated using the bootstrapped standard error to calculate critical ratios for all estimated individual paths to determine significant (*p* < .05; Kline, 2015).

## Results

First, the Brief-COPE and its 14 subscales were explored using EFA. Factor loadings for the 14 subscales of the Brief-COPE are presented in Table 1. To determine the best factor structure, the eigenvalues, factor loadings, scree plot and conceptual character of the factors were taken into account (Ledesma et al., 2015). Based on these criteria, the EFA analysis identified a higher-order four-factor structure accounting for 55.92% of the total variance: (1) Social Support, 22.52%; (2) Positive Coping, 14.25%; (3) Avoidance, 9.90%; and (4) Humor/Religion, 9.26%. Religion was removed from the model as it did not sufficiently (<0.30) load on any of the four factors. Next, this four-factor structure was confirmed using SEM. To obtain a good model fit, two error residuals were correlated. Adequate global model fit, with CFI = 0.92, RMSEA = 0.05, and SRMR = 0.08 and good local model fit, with *ps* < .001, was achieved. Construct reliability validity was evaluated and revealed acceptable Joreskog rho scores: Social Support = .975, Positive Coping = 0.720, Avoidance = 0.735, and Humor = 0.895 (Kline, 2015).

**Table 1.** Factor Loadings for the 14 Subscales of the Brief-COPE.

	Component			
	1	2	3	4
Emotional support	0.844			
Instrumental support	0.786			
Venting	0.764			
Self-distraction		0.583		
Self-blame		0.544		
Denial		0.519		
Behavioral disengagement		0.414		
Substance use		0.311		
Active coping			0.742	
Positive thinking			0.659	
Planning			0.520	
Acceptance			0.494	
Humor				0.428
Religion				

Extraction Method: Principal Axis Factoring. Rotation Method: Oblimin with Kaiser Normalization.

**Figure 1.**
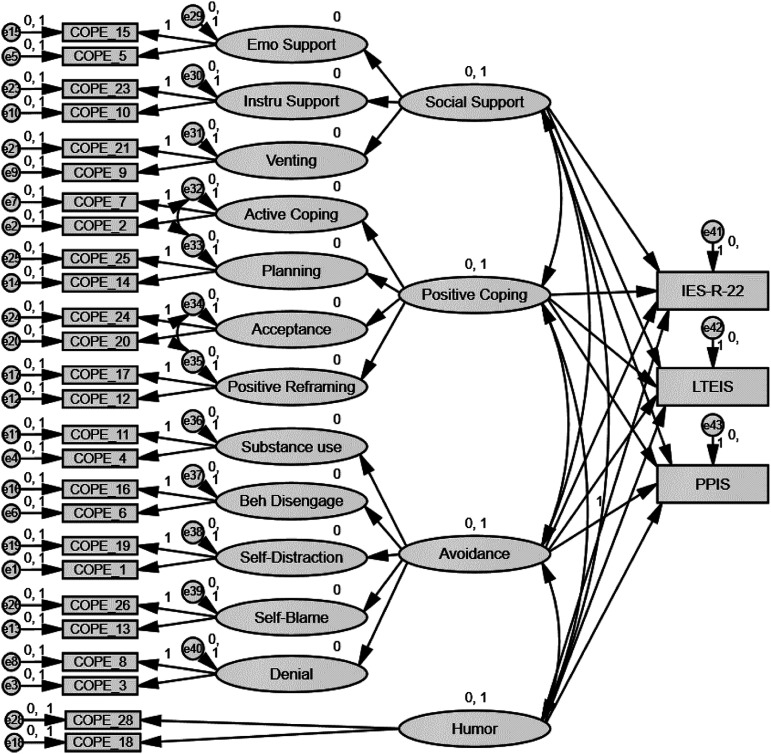
Structural equation modeling analysis for the short-term (IES-R), long-term emotional (LTEIS), and long-term professional (PPIS) impact of client suicide as predicted by the Brief-COPE*.*

Second, a predictive model in SEM was constructed to evaluate the extent to which the four-factor model of the Brief-COPE was able to predict short-and long-term impact (see Table 2, Figure 1). The four-factor model explained 51% of short-term, 64% of long-term emotional and 55% of long-term professional impact. Social Support did not predict any of the impact variables (*ps* > 0.05). Positive Coping had a negative significant predictive value for long-term emotional (b* = −0.27, *p* = .045), but not short-term (b* = −0.07, *p* = .594) or long-term professional (b*= −0.24, *p* = .068) impact. Avoidance had a positive significant predictive value for short-term (b* = 0.70, *p* < .001), long-term emotional (b* = 0.83, *p* < .001) and long-term professional (b* = 0.76, *p* < .001) impact. Lastly, Humor had a negative significant predictive value for short-term (b* = −0.33, *p* < .001), long-term emotional (b* = −0.24, *p* = .004) and long-term professional (b* = −0.25, *p* = .002) impact.

**Table 2.** Regression Weights for the Four Latent Variables in Predicting Short- and Long-Term Outcomes of Client Suicide.

		Unstandardized Estimate	Standardized Estimate	*p*-Value
Social support	IES-R	1.401	0.073	.490
Social support	LTEIS	0.095	0.104	.735
Social support	PPIS	0.131	0.181	.113
Positive coping	IES-R	−1.249	−0.065	.594
Positive coping	LTEIS	−0.246	−0.270	**.045**
Positive coping	PPIS	−0.173	−0.239	.068
Avoidance	IES-R	13.488	0.702	**<.001**
Avoidance	LTEIS	0.756	0.827	**<.001**
Avoidance	PPIS	0.550	0.760	**<.001**
Humor	IES-R	−6.272	−0.327	**<.001**
Humor	LTEIS	−0.218	−0.239	**.004**
Humor	PPIS	−0.179	−0.248	**.002**

*Note*. IES-R = impact of event scale-revised; LTEIS = long-term emotional impact scale; PPIS = professional practice impact scale

## Discussion

### Coping with Loss

The current study aimed to investigate the role of coping strategies in the short- and long-term impact of client suicide and, by doing so, explain individual differences in bereavement. A four-factor model, with Social Support, Positive Coping, Avoidance, and Humor as latent variables, explained 51% of short-term, 64% of the long-term emotional, and 55% of the long-term professional differences in impact. Moreover, while Avoidance positively predicted all three impact outcomes, Positive Coping and Humor negatively predicted one or more impact outcomes, whereas Social Support did not predict impact. Taken together, these results suggest that one’s coping style, in particular Avoidance, Humor, and Positive Coping, is important in understanding individual differences in the impact of client suicide.

Avoidance involves coping strategies such as substance use, self-distraction, behavioral disengagement, self-blame, and denial. Avoidant coping strategies are not considered “bad” coping strategies in themself yet, are generally considered less adequate as a long-term coping style. That being said, our results do indicate that MHPs who rely on avoidant coping styles reported more short- and long-term impact of client suicide. This suggests that, when facing a loss such, reverting to self-blame, guilt trips, substance use or disengagement might not prove helpful in dealing with the emotions, feelings, and thoughts related to loss. Previous research regarding the role of avoidant coping in light of (complicated) grief or bereavement seems to support the current study’s these findings. Eisma et al. (2013), who investigated the relationship between rumination and symptoms of complicated grief and depression following a loss, found that cognitive and experiential avoidance mediates the relationship between grief-specific rumination and distress. In other words, rumination during bereavement increased and perpetuated symptoms of psychopathology. In line with that, Schnider et al. (2007), who investigated coping styles and how they relate to post-traumatic stress and complicated grief, found avoidant coping styles to be positively associated with complicated grief and PTSD severity. Buckley et al. (2015) investigated coping responses and the intensity of bereavement following a death in a critical care environment and found self-blame and denial, part of our *Avoidance* latent variable, to be associated with higher bereavement intensity at 6-month follow-up. Interestingly and contrary to our results, Buckley and colleagues also found the use of emotional support to be associated with higher bereavement intensity at 6-month follow-up. Taken together, one could conclude high levels of avoidant coping are likely to be unhelpful in dealing with client suicide, both in the long and short run.

Positive Coping involves coping strategies like active coping, planning, acceptance, and positive reframing. Again, such strategies are not considered “good” or “bad” coping per se, they focus on taking an active stance or with a positive mindset re-evaluating the issue at hand. Our results indicate that MHPs who employ more positive coping strategies reported less long-term emotional impact of client suicide, however, revealed no significant effect for short-term or long-term professional impact. Previous research regarding the role of active coping, planning, acceptance, and positive reframing concerning (complicated) grief or bereavement appears to be largely in line with our current findings. Riley et al. (2007), who investigated grief responses following the death of a child, found positive reframing, part of our *Positive Coping* latent variable, to be associated with less intense grief reactions and less complicated grief. In line with this, Moore et al. (2010) found positive reframing important in understanding variance in post-trauma functioning among veterans in mental health treatment. Lambert et al. (2012) conducted a series of studies to test if gratitude is related to fewer depressive symptoms through positive reframing and positive emotion. This study found both positive reframing and positive emotion mediated the relationship between gratitude and depressive symptoms. Regarding acceptance, Vujanovic et al. (2009) investigated the relationship between mindfulness-based acceptance and PTSS symptoms among trauma-exposed adults. Their results suggest “accepting without judgment” is associated with lower post-traumatic stress symptoms, regardless of negative affectivity and number of traumas. Equally, Thompson et al. (2011) examined how mindfulness- and acceptance-based theories of psychopathology relate to risk of and resilience to PTSD following trauma. Their results indicate that acceptance is associated with greater psychological adjustment following exposure to trauma. Perhaps pointing in the direction of a mechanism, Rayburn et al. (2005) investigated the relationship between trauma, coping, depression, and mental health service and found active coping to be an important predictor of mental health service seeking among traumatized women. The current study’s findings concerning active coping strategies and the impact of client suicide appear to be largely in line with previous research that suggests that active coping helps deal with trauma.

Humor, which involves being able to make fun of or joke about a difficult situation one is facing, negatively predicted the impact of client suicide, meaning MHPs who rely heavily on humor as a coping strategy reported less impact of client suicide. Relying on humor, however, does not mean that those MHPs were able to or wanting to make light of the client suicide itself, rather, that in general, such attitude might help deal with difficult situations and be associated with less short- and long-term impact. Previous research looking into the importance of humor in relation to dealing with stressful events, in general, has yield similar results (Kuiper, 2012; Lefcourt et al., 1995). Research investigating the importance of humor in relation to grief work or bereavement is limited, yet seems to suggest a similar pattern. Booth-Butterfield et al. (2014) investigated the importance of humor during bereavement following the death of a loved one and report that humor, when not related to the specific death context, was associated with greater coping efficacy and also lead to reduced physical and emotional symptoms. Similarly, Lund et al. (2009) investigated the importance of humor, laughter, and happiness in the daily lives of recently bereaved spouses and report that experiencing humor, laughter, and happiness was associated with favorable bereavement adjustments. Moreover, Ong et al. (2004) highlight that humor can be investigated as both a coping resource that one can deploy as a buffer in stressful times, as well as, as indicative of greater emotional resilience or greater capacity to keep positive emotions separate from negative emotions during stress. Overall, the current findings are in line with previous research that suggests that humor can be helpful when dealing with bereavement.

Finally, Social Support, which involves both emotional and instrumental social support as well as religious or spiritual support, did not significantly predict the impact of client suicide. This is noteworthy since most interventions stress the importance of reaching out to others (Kaniasty, 2012 for a review). That said, while our results suggest that the extent to which one typically relies on social support as a coping strategy is not predictive with regard to short- and long-term impact of client suicide, that does not mean that MHPs did not seek social support after or do not benefit from seeking such support. Rather, one’s tendency to rely on social support is not predictive of the impact of client suicide in MHPs. In other words, if people are inclined to seek social support that can be an important part of healing, but that for those who are less inclined, results do not suggest there is a need to push for that as a strategy either. Previous research investigating social support concerning bereavement has found the size, (in)adequacy, or (un)availability of one’s social support network to be a predictor of complicated grief and post-traumatic stress symptoms (Burke et al., 2010) or, in contrast, a positive predictor of post-traumatic growth (Lipp et al., 2009). That said, most research investigating the role of social support networks has looked into the existence, presence, or availability of social support networks rather than the extent to which social support is commonly used as a coping style. Taken together, the current results are consistent with findings of emotional disclosure research in general, indicating that talking about loss and expressing feelings about the loss do not necessarily facilitate coping with bereavement (W. Stroebe et al., 2005a). Although social support coping strategies can be regarded as helpful, our findings support the current consensus that social support neither softens the impact of loss nor appears to accelerate the process of recovery (W. Stroebe et al., 2005b).

### Implications

The current study, which focused on the relationship between one’s general coping style and the impact of client suicide, has clear implications for both research and clinical practice. When we consider a person’s ability to cope with difficulties, one way to think about it is like a personal toolbox. As such, coping tools are what each bereaved person is “equipped” with; a resource they can draw on at a time of need and that gives direction to coping with specific bereavement-related challenges. Everyone, every toolbox in that sense, is different, includes different options, preferences, and possibilities. By investigating the relationship between one’s general coping style and bereavement by client suicide and identifying which coping styles are associated with (mal)adaptive outcomes, we aim to increase our understanding of the core processes and diversity of “healthy” versus “unhealthy” reactions to dealing with client suicide. MHPs that struggle with bereavement following client suicide may experience difficulties in terms of physical and mental health, as well as may be subject to a wide range of additional consequences, including interpersonal difficulties within the family or at the workplace (M. S. Stroebe et al., 2007). In this context, understanding the importance of one’s general coping style can prove helpful in designing effective pre- or postvention initiatives (Folkman, 2001). Insight into one’s general coping style could then guide MHPs towards reducing the negative consequences following client suicide (Skinner et al., 2003). Taking the current study’s findings into account when developing pre-postvention initiatives is of particular importance as current interventions for the bereaved have proven limited in their effectiveness (Doering & Eisma, 2016).

## References

[bibr1-00302228211073213] Alexander D. A. Klein S. Gray N. M. Dewar I. G. Eagles J. M. (2000). Suicide by patients: Questionnaire study of its effect on consultant psychiatrists. BMJ (Clinical Research Ed.), 320(7249), 1571–1574. 10.1136/bmj.320.7249.1571 PMC27400 10845964

[bibr2-00302228211073213] Booth-Butterfield M. Wanzer M. B. Weil N. Krezmien E. (2014). Communication of humor during bereavement: Intrapersonal and interpersonal emotion management strategies. Communication Quarterly, 62(4), 436–454. 10.1080/01463373.2014.922487

[bibr3-00302228211073213] Buckley T. Spinaze M. Bartrop R. McKinley S. Whitfield V. Havyatt J. Roche D. Fethney J. Tofler G. (2015). The nature of death, coping response and intensity of bereavement following death in the critical care environment. Australian Critical Care, 28(2), 64–70. 10.1016/j.aucc.2015.02.003 25801350

[bibr4-00302228211073213] Burke L. A. Neimeyer R. A. McDevitt-Murphy M. E (2010). African American homicide bereavement: Aspects of social support that predict complicated grief, PTSD, and depression. OMEGA - Journal of Death and Dying, 61(1), 1–24. 10.2190/OM.61.1.a 20533646

[bibr5-00302228211073213] Carver C. S. (1997). You want to measure coping but your protocol’s too long: consider the brief COPE. International Journal of Behavioral Medicine, 4(1), 92–100. 10.1207/s15327558ijbm0401_6 16250744

[bibr6-00302228211073213] Carver C. S. Scheier M. F. Weintraub J. K. (1989). Assessing coping strategies: A theoretically based approach. Journal of Personality and Social Psychology, 56(2), 267–283. 10.1037//0022-3514.56.2.267 2926629

[bibr7-00302228211073213] Chemtob C. M. Bauer G. B. Hamada R. S. Pelowski S. R. Muraoka M. Y. (1989). Patient suicide: Occupational hazard for psychologists and psychiatrists. Professional Psychology: Research and Practice, 20(5), 294–300. 10.1037/0735-7028.20.5.294

[bibr8-00302228211073213] Chemtob C. M. Hamada R. S. Bauer G. Kinney B. Torigoe R. Y. (1988). Patients’ suicides: Frequency and impact on psychiatrists. The American Journal of Psychiatry, 145(2), 224–228. 10.1176/ajp.145.2.224 3341466

[bibr54-00302228211073213] Creamer M. Bell R. Failla S. (2003). Psychometric properties of the Impact of Event Scale—Revised. Behaviour Research and Therapy, 41(12), 1489–1496. 10.1016/j.brat.2003.07.010. 14705607

[bibr9-00302228211073213] Darden A. J. Rutter P. A. (2011). Psychologists’ experiences of grief after client suicide: A qualitative study. Omega, 63(4), 317–342. 10.2190/OM.63.4.b 22010371

[bibr10-00302228211073213] Doering B. K. Eisma M. C. (2016). Treatment for complicated grief: State of the science and ways forward. Current Opinion in Psychiatry, 29(5), 286–291. 10.1097/YCO.0000000000000263 27429216

[bibr11-00302228211073213] Dransart DA Gutjahr E Gulfi A Didisheim NK Séguin M (2014). Patient suicide in institutions: Emotional responses and traumatic impact on Swiss mental health professionals. Death Studies, 38(1-5), 315–321. 10.1080/07481187.2013.766651 24593010

[bibr12-00302228211073213] Eagles J. M. Klein S. Gray N. M. Dewar I. G. Alexander D. A. (2001). Role of psychiatrists in the prediction and prevention of suicide: A perspective from north-east Scotland. The British Journal of Psychiatry: The Journal of Mental Science, 178(6), 494–496. 10.1192/bjp.178.6.494 11388963

[bibr13-00302228211073213] Eisma M. C. Stroebe M. S. Schut H. A. W. Stroebe W. Boelen P. A. van den Bout J. (2013). Avoidance processes mediate the relationship between rumination and symptoms of complicated grief and depression following loss. Journal of Abnormal Psychology, 122(4), 961–970. 10.1037/a0034051 24364599

[bibr14-00302228211073213] Ellis T. E. Patel A. B. (2012). Client suicide: What now? Cognitive and Behavioral Practice, 19(2), 277–287. 10.1016/j.cbpra.2010.12.004

[bibr15-00302228211073213] Elmose M. Duch C. Elklit A. (2016). Children’s coping styles and trauma symptoms after an explosion disaster. Scandinavian Journal of Child and Adolescent Psychiatry and Psychology, 4(3), 132–140. 10.21307/sjcapp-2016-020

[bibr16-00302228211073213] Finlayson M. Simmonds J. G. (2018). Impact of client suicide on psychologists in Australia. Australian Psychologist, 53(1), 23–32. 10.1111/ap.12240

[bibr17-00302228211073213] Folkman S. (2001). Revised coping theory and the process of bereavement. In Stroebe M. S. Hansson R. O. Stroebe W. Schut H. (Eds.) Handbook of bereavement research: Consequences, coping and careHandbook of bereavement research: Consequences, coping, and care (pp. 563–584). American Psychological Association. 10.1037/10436-000

[bibr18-00302228211073213] Gaffney M. Hannigan B. (2010). Suicide bereavement and coping: A descriptive and interpretative analysis of the coping process. Procedia - Social and Behavioral Sciences, 5, 526-535. 10.1016/j.sbspro.2010.07.137.

[bibr19-00302228211073213] Greenberg D. Shefler G. (2014). Patient suicide. The Israel Journal of Psychiatry and Related Sciences, 51(3), 193–198. 25618283

[bibr20-00302228211073213] Gulfi A. Castelli Dransart D. A. Heeb J.-L. Gutjahr E. (2010). The impact of patient suicide on the professional reactions and practices of mental health caregivers and social workers. Crisis, 31(4), 202–210. 10.1027/0027-5910/a000027 20801750

[bibr21-00302228211073213] Hendin H. Lipschitz A. Maltsberger J. T. Haas A. P. Wynecoop S. (2000). Therapists’ reactions to patients’ suicides. The American Journal of Psychiatry, 157(12), 2022–2027. 10.1176/appi.ajp.157.12.2022 11097970

[bibr57-00302228211073213] Henry M. Séguin M. Drouin M.-S. (2004). Les réactions des professionnels en snaté mentale au décès par suicide d’un patient. [Mental health professionals’ response to the suicide of their patients.]. Revue Québécoise de Psychologie, 25(3), 241-257.

[bibr56-00302228211073213] Horn P. J. (1994). Therapists’ psychological adaptation to client suicide. Psychotherapy: Theory, Research, Practice, Training, 31(1), 190-195. 10.1037/0033-3204.31.1.190.

[bibr22-00302228211073213] Hu L. T. Bentler P. M. (1999). Cutoff criteria for fit indexes in covariance structure analysis: conventional criteria versus new alternatives. Structural Equation Modeling, 6(1), 1–55. 10.1080/10705519909540118

[bibr23-00302228211073213] Jeavons S. (2000). Predicting who suffers psychological trauma in the first year after a road accident. Behaviour Research and Therapy, 38(5), 499–508. 10.1016/S0005-7967(99)00073-X 10816908

[bibr24-00302228211073213] Kaniasty K. (2012). Predicting social psychological well-being following trauma: The role of postdisaster social support. Psychological Trauma: Theory, Research, Practice, and Policy, 4(1), 22–33. 10.1037/a0021412

[bibr25-00302228211073213] Kleespies P. M. Smith M. R. Becker B. R. (1990). Psychology interns as patient suicide survivors: Incidence, impact, and recovery. Professional Psychology: Research and Practice, 21(4), 257–263. 10.1037/0735-7028.21.4.257

[bibr26-00302228211073213] Kline R. B. (2015). Principles and practice of structural equation modeling (4th ed.). Guilford Publications.

[bibr27-00302228211073213] Kuiper N. A. (2012). Humor and resiliency: Towards a process model of coping and growth. Europe’s Journal of Psychology, 8(3), 475–491. 10.5964/ejop.v8i3.464

[bibr28-00302228211073213] Lambert N. M. Fincham F. D. Stillman T. F. (2012). Gratitude and depressive symptoms: the role of positive reframing and positive emotion. Cognition and Emotion, 26(4), 615–633. 10.1080/02699931.2011.595393 21923564

[bibr29-00302228211073213] Lazarus R. S. Folkman S. (1984). Stress, appraisal, and coping. Springer Publishing Company.

[bibr30-00302228211073213] Ledesma R. D. Valero-Mora P. Macbeth G. (2015). The scree test and the number of factors: A dynamic graphics approach. The Spanish Journal of Psychology, 18, E11. 10.1017/sjp.2015.13. 26055575

[bibr31-00302228211073213] Lefcourt H. M. Davidson K. Shepherd R. Phillips M. Prkachin K. Mills D. (1995). Perspective-taking humor: Accounting for stress moderation. Journal of Social and Clinical Psychology, 14(4), 373–391. 10.1521/jscp.1995.14.4.373

[bibr32-00302228211073213] Lipp O. V. Price S. M. Tellegen C. L. (2009). No effect of inversion on attentional and affective processing of facial expressions. Emotion (Washington, D.C.), 9(2), 248–259. 10.1037/a0014715 19348536

[bibr33-00302228211073213] Lund D. A. Utz R. Caserta M. S. de Vries B. (2009). Humor, laughter, and happiness in the daily lives of recently bereaved spouses. OMEGA - Journal of Death and Dying, 58(2), 87–105. 10.2190/OM.58.2.a PMC2646184 19227000

[bibr34-00302228211073213] Moore S. A. Varra A. A. Michael S. T. Simpson T. L. (2010). Stress-related growth, positive reframing, and emotional processing in the prediction of post-trauma functioning among veterans in mental health treatment. Psychological Trauma: Theory, Research, Practice, and Policy, 2(2), 93–96. 10.1037/a0018975

[bibr35-00302228211073213] Ong A. D. Bergeman C. S. Bisconti T. L. (2004). The role of daily positive emotions during conjugal bereavement. The Journals of Gerontology: Series B, 59(4), P168–P176. 10.1093/geronb/59.4.P168 15294920

[bibr36-00302228211073213] Rayburn N. R. Wenzel S. L. Elliott M. N. Hambarsoomians K. Marshall G. N. Tucker J. S. (2005). Trauma, depression, coping, and mental health service seeking among Impoverished women. Journal of Consulting and Clinical Psychology, 73(4), 667–677. 10.1037/0022-006X.73.4.667 16173854

[bibr37-00302228211073213] Riley L. P. LaMontagne L. L. Hepworth J. T. Murphy B. A (2007). Parental grief responses and personals growth following the death of a child. Death Studies, 31(4), 277–299. 10.1080/07481180601152591 17378106

[bibr38-00302228211073213] Rothes I. A. Scheerder G. Van Audenhove C. Henriques M. R. (2013). Patient suicide: The experience of flemish psychiatrists. Suicide & Life-Threatening Behavior, 43(4), 379–394. 10.1111/sltb.12024 23530711

[bibr39-00302228211073213] Sanders S. Jacobson J. M. Ting L. (2008). Preparing for the Inevitable: Training social workers to cope with client suicide. Journal of Teaching in Social Work, 28(1–2), 1–18. 10.1080/08841230802178821

[bibr40-00302228211073213] Savalei V. (2018). On the computation of the RMSEA and CFI from the mean-and-variance corrected test statistic with nonnormal data in SEM. Multivariate Behavioral Research, 53(3), 419–429. 10.1080/00273171.2018.1455142 29624085

[bibr41-00302228211073213] Savalei V. (2021). Improving fit Indices in structural equation modeling with categorical data. Multivariate Behavioral Research, 56(3), 390–407. 10.1080/00273171.2020.1717922 32054327

[bibr42-00302228211073213] Schnider K. R. Elhai J. D. Gray M. J. (2007). Coping style use predicts posttraumatic stress and complicated grief symptom severity among college students reporting a traumatic loss. Journal of Counseling Psychology, 54(3), 344–350. 10.1037/0022-0167.54.3.344

[bibr43-00302228211073213] Skinner E. A. Edge K. Altman J. Sherwood H. (2003). Searching for the structure of coping: A review and critique of category systems for classifying ways of coping. Psychological Bulletin, 129(2), 216–269. 10.1037/0033-2909.129.2.216 12696840

[bibr44-00302228211073213] Stratta P. Capanna C. Dell’Osso L. Carmassi C. Patriarca S. Di Emidio G. Riccardi I. Collazzoni A. Rossi A. (2015). Resilience and coping in trauma spectrum symptoms prediction: A structural equation modeling approach. Personality and Individual Differences, 77, 55-61. 10.1016/j.paid.2014.12.035.

[bibr45-00302228211073213] Stroebe M. S. Schut H. Stroebe W. (2007). Health outcomes of bereavement. Lancet (London, England), 370(9603), 1960–1973. 10.1016/S0140-6736(07)61816-9 18068517

[bibr46-00302228211073213] Stroebe W. Schut H. Stroebe M. S. (2005a). Grief work, disclosure and counseling: do they help the bereaved? Clinical Psychology Review, 25(4), 395–414. 10.1016/j.cpr.2005.01.004 15914264

[bibr47-00302228211073213] Stroebe W. Zech E. Stroebe M. S. Abakoumkin G. (2005b). Does social support help in bereavement? Journal of Social and Clinical Psychology, 24(7), 1030–1050. 10.1521/jscp.2005.24.7.1030

[bibr48-00302228211073213] Thompson R. W. Arnkoff D. B. Glass C. R. (2011). Conceptualizing mindfulness and acceptance as components of psychological resilience to trauma. Trauma, Violence, & Abuse, 12(4), 220–235. 10.1177/1524838011416375 21908440

[bibr49-00302228211073213] Ting L. Sanders S. Jacobson J. M. Power J. R. (2006). Dealing with the aftermath: A qualitative analysis of mental health social workers’ reactions after a client suicide. Social Work, 51(4), 329–341. 10.1093/sw/51.4.329 17152631

[bibr50-00302228211073213] Tyupa S. (2011). A theoretical framework for back-translation as a quality assessment tool. New Voices in Translation Studies, 7, 35-46. https://ruj.uj.edu.pl/xmlui/handle/item/15068.

[bibr51-00302228211073213] Vujanovic A. A. Youngwirth N. E. Johnson K. A. Zvolensky M. J. (2009). Mindfulness-based acceptance and posttraumatic stress symptoms among trauma-exposed adults without axis I psychopathology. Journal of Anxiety Disorders, 23(2), 297–303. 10.1016/j.janxdis.2008.08.005 18834701 PMC2655122

[bibr52-00302228211073213] Wang S. Chen C.-C. Dai C.-L. Richardson G. B. (2018). A call for, and beginner’s guide to, measurement invariance testing in evolutionary psychology. Evolutionary Psychological Science, 4(2), 166–178. 10.1007/s40806-017-0125-5

[bibr55-00302228211073213] Weiss D. S. Marmar C. R. (1997). The Impact of Event Scale—Revised. In Assessing psychological trauma and PTSD (pp. 399-411). The Guilford Press.

[bibr53-00302228211073213] Xia Y. Yang Y. (2019). RMSEA, CFI, and TLI in structural equation modeling with ordered categorical data: The story they tell depends on the estimation methods. Behavior Research Methods, 51(1), 409–428. 10.3758/s13428-018-1055-2 29869222

